# Antioxidant Properties and Redox-Modulating Activity of Chitosan and Its Derivatives: Biomaterials with Application in Cancer Therapy

**DOI:** 10.1089/biores.2019.0028

**Published:** 2020-03-12

**Authors:** Donika G. Ivanova, Zvezdelina L. Yaneva

**Affiliations:** Department of Pharmacology, Animal Physiology and Physiology Chemistry, Trakia University, Stara Zagora, Bulgaria.

**Keywords:** cancer, chitosan, natural products, reactive oxygen species (ROS)

## Abstract

Many studies have shown that mitochondrial metabolism has a fundamental role in induction of carcinogenesis due to the influence of increased levels of reactive oxygen species (ROS) generation in all steps of oncogene transformation and cancer progression. It is widely accepted that the anticancer effect of conventional anticancer drugs is due to induction of oxidative stress and elevated intracellular levels of ROS, which alter the redox homeostasis of cancer cells. On the other hand, the harmful side effects of conventional anticancer chemotherapeutics are also due to increased production of ROS and disruption of redox homeostasis of normal cells and tissues. Therefore, there is a growing interest toward the development of natural antioxidant compounds from various sources, which could impact the redox state of cancer and normal cells by different pathways and could prevent damage from oxidant-mediated reactions. It is known that chitosan exhibits versatile biological properties, including biodegradability, biocompatibility, and a less toxic nature. Because of its antioxidant, antibacterial, anticancer, anti-inflammatory, and immunostimulatory activities, the biopolymer has been used in a wide variety of pharmaceutical, biomedical, food industry, health, and agricultural applications and has been classified as a new physiologically bioactive material.

## Introduction

Normally in the cells, due to their metabolism, reactive oxygen species (ROS), such as superoxide radical, hydroxyl radical, hydroperoxyl radical, and hydrogenperoxide, have been produced.^1^ ROS are generated in mitochondrion, during mitochondrial electron transport in the electron-transport chain,^1–4^ by nicotinamide adenine dinucleotide phosphate (NADPH)-dependent oxidase^4,5^ and by existing enzyme processes such as activation of cytochrome P450,^1,6^ xanthine oxidase,^1,7,8^ as well as other intracellular sources. The level of physiologically generated ROS is controlled by enzymatic and nonenzymatic intracellular antioxidant systems, which are connected with different functional pathways.^8,9^ The cellular oxidative stress is a result of redox imbalance due to enhancement of ROS or suppression and crash of antioxidant systems.^1,8–10^

Overproduction of ROS could be also initiated by extracellular sources such as sunlight, ultraviolet light, chemical reactions, and the impact of different xenobiotics as well as chemotherapies drugs.^11^ The production of ROS above permissible levels could result in damage of cellular macromolecules, for example to change the structure of DNA, membrane lipids, and proteins, which may result in diseases such as cancer, aging, inflammation, and different neurodegenerative disorders as well as other diseases.^2,4,9,11^ Therefore, there has been a growing interest in the development of natural antioxidant compounds from various sources, which could impact the redox state of the cells and could prevent damage from oxidant-mediated reactions.

Chitosan is the deacetylated form of chitin, a biopolymer that occurs naturally as a component of fungal cell walls, insect exoskeletons, and crustacean shells. Chemically, it is a linear copolymer consisting of β-(1 → 4)-2-acetamido-d-glucose and β-(1 → 4)-2-amino-d-glucose units. It is known that it exhibits versatile biological properties, including biodegradability, biocompatibility, and a less toxic nature.^12,13^ As a biomaterial, chitosan elicits a negligible immune response, after implantation, injection, topical application, or ingestion in the mammalian system.^14,15^

The natural cationic polysaccharide possesses attractive hemostatic properties. It has been reported that chitosan stimulates the host immune system against viral and bacterial infection.^13,16,17^ Because of its biological properties, including antioxidant, antibacterial, anticancer, anti-inflammatory, and immunostimulatory activities, the biopolymer has been used in a wide variety of pharmaceutical, biomedical, food industry, health, and agricultural applications and has been classified as a new physiologically bioactive material.^13,18–23^

In this mini-review, we will focus on the antioxidant properties, redox-regulatory activities of chitosan and its derivatives, and their application as anticancer agents.

## Antioxidant Activities of Chitosan and Its Derivatives

Antioxidant activity is one of the well-known functions of chitosan and due to that reason the biopolymer and its derivatives have attracted the attention of researchers from different research areas.^24,25^ Many *in vitro* and *in vivo* studies have shown that chitosan exhibits redox-regulatory activity due to inhibition of ROS production, prevention of lipid oxidation by significantly reduced serum free fatty acids, and malondialdehyde concentrations and it increases intracellular antioxidant enzymes in biological systems. Santhosh et al. demonstrated the prevention of oxidation of hepatotoxic lipids by chitosan, when it was administrated in rats treated with isoniazid or rifampicin.^26,27^

Other experiments with rats reported that chitosan inhibits glycerol-induced renal oxidative damage.^26,28^ Xie et al. reported that the scavenging of hydroxyl radicals by chitosan inhibits the lipid peroxidation of phosphatidylcholine and linoleate liposomes.^26,29^ Cho et al. studied the antioxidant properties of plain chitosan on RAW264,7 mouse macrophage cells. They have demonstrated that plain chitosan in concentration up to 500 μg/mL did not exert a toxic effect on this cell line, but significant lipid peroxidation inhibition by scavenging the lipid-derived radicals was reported.^30^

Wen et al. presented a comparative study about activation of intracellular antioxidant enzymes by chitosan, chitosan nanoparticles, and vitamin C on mouse macrophages RAW264,7 cell line.^26^ They established that treatment of RAW264,7 cells with 500 μM H_2_O_2_ for 12 h caused a decrease in superoxide dismutase (SOD) and GSH activity, but preincubation with chitosan nanoparticles at 100 μg/mL restored the H_2_O_2_-induced decrease of the antioxidant activity. Moreover, the data showed that chitosan nanoparticles restored more effectively SOD and GSH activity than plain chitosan, and the recovery of the antioxidant activity was close to the recovery induced by vitamin C at 250 μM/mL. The authors assumed that the protective mechanism of action of chitosan nanoparticles against H_2_O_2_-induced RAW264,7 cell injury was due to restoring the activities of endogenous antioxidants, along with enhancement of their gene expression.^26^

There are also observations that chitosan can scavenge free radicals or chelate metal ions from the donation of hydrogen or the lone pairs of electrons.^24,28,29^ The fact that chitosan shows a strong metal-ion chelating ability suggests that it could be a potential natural product antioxidant, especially when the deactivation of catalytic activity of metal ions, for example Fe^2+^/Fe^3+^, was considered^31,32–34^ These metals, which are a key aspect of effective antioxidant systems, represent the main catalysts of oxidation processes in biological systems, because they participate in the oxidation of cells macromolecules, such as of lipids, DNA, etc.^20,21,35–37^ The interaction of chitosan with metal ions could involve several complex processes, including adsorption, ion exchange, and chelation.^38^

In relation to the cited experimental studies of chitosan, several mechanisms about chitosan antioxidant action have been proposed.^24^ The hydroxyl (-OH) and amino (-NH_2_) groups in chitosan are the key functional groups for its antioxidant activity, but their dissociation could be difficult, due to the semi-crystalline structure of chitosan and the presence of strong hydrogen bonds.^29^ On the other hand, there are studies that have shown that to develop its antioxidant activities, chitosan has to overcome: (1) poor solubility, (2) chemical inertness based on the strong inter- and intramolecular hydrogen bonds network, and (3) poor H-atom-donating ability to serve as a good chain-breaking antioxidant.^31^

To confirm these allegations, Alexandrova et al. established that the antioxidant activity of chitosan was essentially zero.^31,39^ Another study of Li et al. reported the very high EC_50_(1.2 × 10^6^ μgmL^−1^) of chitosan from EPR studies, pointing out that inter- and intramolecular hydrogen bonds blunt free-radical reactions.^31,40^ To enhance water solubility of chitosan by disrupting inter- and intramolecular hydrogen bonding, a number of researchers proposed the synthesis of chitosan derivatives with improved solubility. Their antioxidant activities were proved by experiments conducted in both cell free^13,31,41–44^ and cell environment.

For example, Ngo et al. examined the antioxidant capacity of chitosan gallate on SW1353 cells and on mouse macrophage exposed to H_2_O_2_.^45^ The data from both studies indicated an increase in the intracellular antioxidant enzymes (SOD and GSH), suppression of the NF-kB activity, and prevention of oxidative damage to cellular biomolecules in living cells by both indirect and direct ways.^45^ Liu et al. used ECV304 cells that were pretreated with different concentrations of chitooligosaccharides before exposure to H_2_O_2_. They have observed statistically significant suppression of the intracellular levels of ROS by chitooligosaccharides in the concentration range of 100–200 g/mL. They established that the effects of chitooligosaccharides as ROS-scavenging agents were similar to those induced by vitamin C in a concentration of 250 g/mL, and they also demonstrated a significant decrease of malondialdehyde at the intracellular level (a marker of lipid peroxidation).^46^

A number of studies indicated that chitooligosaccharides regulated the antioxidant enzyme activities due to elevated expression of intracellular antioxidant enzymes, such as SOD, catalase (CAT), glutathione reductase (GR), and glutathione peroxidase (GPx) under H_2_O_2_-mediated oxidative stress on both rats and cell cultures.^26,47–51^ For example, Mendis et al. studied the redox modulation capacity of chitooligosaccharides in different concentrations on the B16F1 cell line and established that different molecular weight oligosaccharides exhibited differential capabilities to increase cellular GSH levels, due to different permeability of chitooligosaccharides into the cells.^52^

Similar results of intracellular antioxidant enzyme activation by chitooligosaccharides have been observed by Liu et al. They described that the preincubation of ECV304 cells with 300 μM H_2_O_2_ and treatment with chitooligosaccharides at a concentration 100 μg/mL restored the activity of the antioxidant enzymes SOD and GSH by about 44.9% and 23.5%, respectively. The authors compared the antioxidant activity of chitooligosaccharides with that of vitamin C in a concentration of 250 μg/mL and observed that at the same working conditions, SOD and GSH were restored by vitamin C with 19.2% and 15.1%, respectively.^46^ The experimental data of Xu et al. and Luo et al. proved that chitooligosaccharides (0.1–1 mg/mL) alleviated oxidative stress-associated cellular damage and induction of apoptosis in hepatocytes via mechanisms requiring the NF-E2-related factor-2 mediated upregulation of antioxidant enzymes, including heme oxygenase-1, NAD(P)H, quinone dehydrogenase-1, and SOD.^53–55^

With respect to the antioxidant properties of chitosan and its derivatives, the review data outlined a number of factors that may affect their antioxidant activity, such as molecular weight and deacetylation degree of chitosan, the grafting method, and the grafting ratio.^56^ Besides, it could be stated that chitosan and its derivatives possess redox-regulatory activity, because they could act as scavenger cellular-free radicals and, subsequently, to inhibit radical mediated cellular oxidation, which could, in turn, lead to restoration of intracellular antioxidant enzyme activities and, consequently, to restoration of the normal functioning of oxidation biochemical processes in the cells.

## Anticancer Effects of Chitosan and Its Derivatives

Carcinogenesis is a multistep process, accompanied by accumulated genetic alterations in the somatic cells.^56,57^ However, different studies have shown that mitochondrial metabolism has a fundamental role in uncontrolled cell proliferation because many scientific researchers have demonstrated the influence of increased levels of ROS generation in all steps of oncogene transformation and cancer progression.^58–61^ Many studies suggest that due to their redox metabolism, cancer cells are characterized with increased ROS generation, as well as over-expression of antioxidant enzymes in response to the permanent oxidative stress, in comparison to normal cells.^13,62–66^ But at the same time, there are many evidences that have shown that mitochondria and producing ROS are key regulators for induction of cancer cell death.^67–72^

**FIG. 1.**
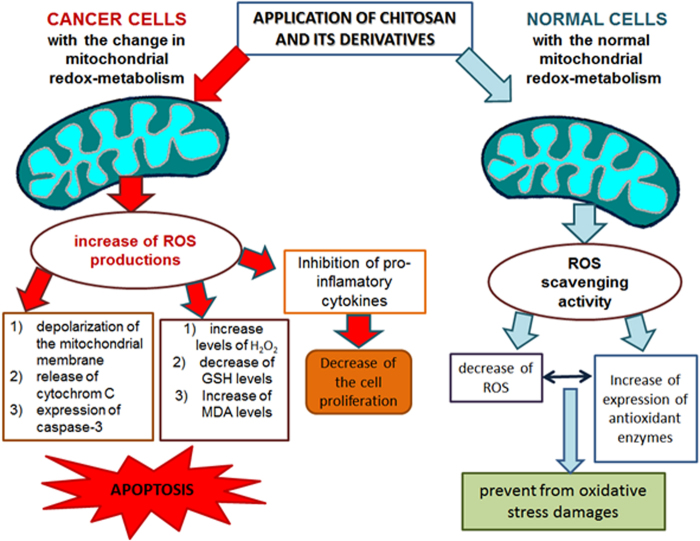
Influence of redox-regulatory activity of chitosan and its derivatives on cell viability of both cancer and normal cells.

Natural products have played a pivotal rule in guiding researchers to develop efficient anticancer agents.^73^ Many polysaccharides extracted from natural sources have been found to possess a variety of biological activities and can be classified into two groups based on their sources, natural and semi-synthetic polysaccharides, which are produced by chemical or enzymatic modifications of the parent macromolecules.^74^ In recent years, numerous studies have reported similar natural compounds with redox-modulation activity, when applied in cancer treatment and in the same time, without any cytotoxic effect, when it is applied on normal cells. There are confirmatory studies that have demonstrated the anticancer and chemo-preventive effect of chitosan and its derivatives on cancer cells and no effect on normal cell viability. The data are summarized in Table 1. It was proposed that the different effect on cancer and normal cells was due to redox-regulation activity of chitosan (Fig. 1).

**Table 1.** Some of the Most Relevant Findings That Described Different Activity of Chitosan and Its Derivatives on Normal and Cancer Cells (Redox-Regulatory Activity)

Type chitosan	Type cells	Observed activity	Ref.
Antioxidant capacity			
Chitosan nanoparticles (at concentration 100 μg/mL)	RAW264,7 cells exposed to 500 μM H_2_O_2_ for 12 h incubation time	Protective mechanism, due to restoration of H_2_O_2_-induced decrease of activity of SOD and GSH. The recovery of antioxidant enzyme activity in the cells was close to the recovery induced by vitamin C at a concentration of 250 μM/mL; enhancement of enzyme gene expression.	^26^
Chitosan gallate (at concentration up to 100 μg/mL)	SW1353 and mouse macrophage, exposed to 2 mM H_2_O_2_ and 0.1 M FeSO_4_ (Fenton reaction) and incubation time of 30 min	Nontoxic effect; increase of intracellular SOD and CAT activity on both studies; suppression of NF-kB activity; prevention of oxidative damage by indirect and direct ways.	^66^
Chitooligosaccharides (in concentration range of 25–200 g/mL)	ECV304 cells exposed to H_2_O_2_ in a concentration of 300 μM and incubation time of 12 h	Preventive effect on suppressing the production of lipid peroxidation; restored activity of endogenous antioxidants (SOD and GSH-Px); decrease of intracellular ROS level production and ROS-scavenging activity, which were commensurable with antioxidant activity of vitamin C, applied in a concentration of 250 g/mL.	^67^
Anticancer activity			
Chitosan (in concentration up to 500 μg/mL^−1^ and incubation time of 24, 48, and 72 h)	MDA-MB-231, MCF-7, T47D breast cancer cells	Inhibitory effect on cell proliferation; depolarization on mitochondrial membrane; increased ROS production, DNA oxidation, and S phase cell cycle arrest.	^65^
Chitosan nanoparticles (at a dose of 0.5 mg/kg body weight for a period of 15 consecutive days)	Female mice bearing solid Ehrlich carcinoma in neck region	Antitumor activity; increased malonedialdehyde levels (marker for lipid peroxidation) and decreased GSH levels.	^77^
Chitosan-copper complex (0.5 g chitosan was dissolved in 50 mL of 1% acetic acid solutions containing different amounts of copper sulfate)	Tumor cell lines 293 and HeLa and normal lung fibroblast cell line HLF	Inhibition of tumor cell line proliferation, but not that of the normal human lung fibroblast cell line HLF.	^7^

ROS, reactive oxygen species; SOD, superoxide dismutase.

Salehi et al. observed that chitosan exerts an inhibitory effect on the proliferation of MDA-MB-231, MCF-7, and T47D breast cancer cells in a dose- and time-dependent manner, while being non-toxic to fibroblast L929 normal cells. They have shown that exposure of MDA-MB-231 cells to chitosan led to depolarization of the mitochondrial membrane, increase of ROS generation, DNA oxidation, and S phase cell cycle arrest. The authors also established alteration expression of caspase 3, which indicated that MDA-MB-231 cells become progressively apoptotic on chitosan exposure.^75^

Dou et al. investigated that chitooligosaccharides at a concentration of 100 mg/mL applied on neutrophils from glycogen-induced peritonitis mice caused the production of superoxide and H_2_O_2_, as well as induction of apoptosis. Moreover, SOD administration could abolish the proapoptotic effect induced by chitooligosaccharides. The authors observed that increased production of superoxide in neutrophils by chitooligosaccharides has a key role in induction of apoptosis in these cells. They also demonstrated that the addition of inhibitors of phospholipase D and the PI3K signaling pathway suppressed generation of superoxide, which lead to the assumption that production of superoxide induced by chitooligosaccharides is due to activation of phospholipase D and the PI3K signaling pathway.^76^

In the study of Ahmed et al., the effect of chitosan nanoparticles on tumor neovascularization growth on the model of female mice bearing solid Ehrlich carcinoma in the neck region was presented. The authors observed that *in vitro*, chitosan nanoparticles showed high antitumor activities, which were accompanied with an increase in MDA level and a decrease in GSH level in tumor tissues.^77^

Martínez-Torres et al. established that chitosan gold nanoparticles (3–10 nm) are cytotoxic in a dose-dependent manner in cervical (HeLa) and breast (MCF-7) cancer cell lines. Incubation of peripheral blood mononuclear cells with chitosan gold nanoparticles in the same conditions displayed induction of low cytotoxicity in these cells. The cell death mechanism is specific for the type of cancer cell line tested, but in all cases, ROS production is mandatory for cell death induction by chitosan gold nanoparticles. The experimental results demonstrated that inhibition of ROS production with N-acetyl cysteine leads to inhibition of cell death.^78^

Zheng et al. investigated the effect of chitosan-copper complexes on tumor cell lines 293 and HeLa and normal lung fibroblast cell line HLP. After 48 h of incubation time, cell proliferation was investigated and the results indicated that chitosan-copper complexes selectively inhibited HeLa and 293 tumor cell line proliferation, but there was no inhibition in the growth of HLF.^79^ Another research observed that chitooligosaccharides (a polymerization degree of 2–8) could decrease tumor angiogenesis and exhibited antioxidant activity by augmenting the SOD activity in Kunming mice that were implanted with human breast cancer cells, dose dependently.^80^

Chemo-preventive activity of chitooligosaccharides in human colorectal adenocarcinoma cells line (HT-29) was reported to be the result of regulation activity of intracellular antioxidant enzymes GSH and GR.^81^ It was also observed that chitooligosaccharides inhibited proinflammatory cytokine-mediated nitric oxide (NO^•^) production and inducible NO synthase (iNOS), leading to a decrease in proliferation of HT-29.^82^

## The Redox Activity of Chitosan: A Possible Mechanism for Sensitizing Cancer Cells Toward Chemotherapeutics

**FIG. 2.**
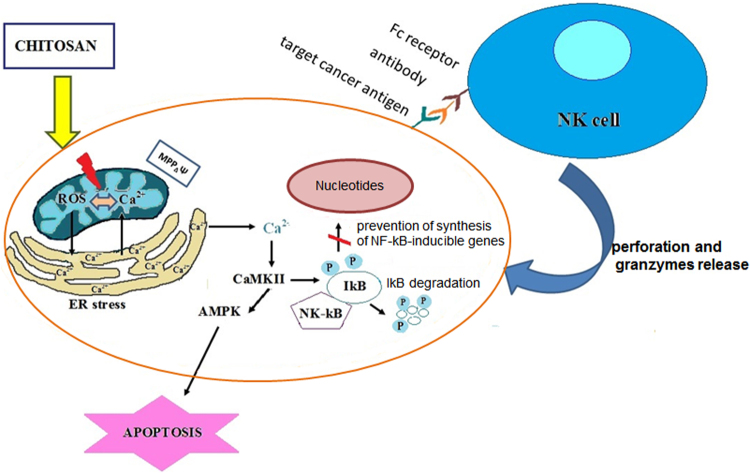
A possible specific cancer cell mechanism, for elevation of intracellular ROS production, which could be closely associated with activation of calcium signalization and enhancement of the human defense system. ROS, reactive oxygen species.

The redox-regulatory mechanisms of chitosan described earlier as well as other research results could be used as a reason for conducting a deeper study about anti-carcinogenic mechanisms of chitosan. There are data that have shown that the initiation by chitosan intracellular elevation of ROS generations, specifically in cancer cells, could be closely linked with activation of intracellular calcium signalization and lead to enhancement of the human defense system (Fig. 2).

There are evidences that have demonstrated that the human immune system has great potential to destroy cancer cells without being toxic to the healthy tissue and organs and the activation and effector role of immune cells is dependent on Ca^2+^ influx.^83–89^ The distinct immune cells are able to recognize cancer cells by forming Ca^2+^-dependent cytotoxicity and using a killing mechanism either through the release of lytic granules and granzymes or by activation of the Fas-Fas ligand receptor, known as the death receptor.^83–89^ Further, it has recently been shown that increased Ca^2+^ concentration in the cytosol is crucial for lytic granule exocytosis in natural killer cells and CTLs as well as production of cytokines (TNF-α and IFN-γ) by natural killer cells.^87^ It has been also demonstrated that along with the depolarizing nature of cancer cells, Ca^2+^ concentration can also be a marker of the action of killer T cells.^86,89^

In this respect, it is suggested that the antitumor activity of water-soluble chitosan might be related, in part, to an enhancement of the proliferation of cytolytic lymphocytes, natural killer cells.^90,91^ Among the first who reported about immune enhancement by chitosan are Suzuki et al. They proved that the antitumor mechanism of chitooligosaccharides is to enhance acquired immunity by accelerating T cell differentiation to increase cytotoxicity and maintain T cell activity.^92^

Using both *in vitro* and *in vivo* models, many scientific researchers have shown that chitooligosaccharides do hold promise in boosting both the innate and adaptive immunities.^93^ The dose ranges of chitooligosaccharides that have been found to produce the immunostimulating effects are 10–100 μg/mL for *in vitro* studies and 100–500 mg/kg/day for *in vivo* studies.^94,95^ It was observed that within these concentration ranges chitooligosaccharides stimulated the secretion of TNF-α and IL-1β from macrophages and increased iNOS expression and NO^•^ production in macrophages, which led to enhancement of the tumor-killing ability of macrophages.^94–96^ It is well known that NO^•^ is produced by nitric oxide synthase, of which two isoforms (nNOS/NOS1 and eNOS/NOS3) are regulated in a Ca^2+^-calmodulin-dependent manner.^97,98^

On the other hand, there are evidences that have shown that chitooligosaccharides applied on both colorectal cancer cell lines and colitis-associated colorectal cancer in mice lead to suppression of cancer development by stimulation of AMP-activated protein kinase (AMPK), increase of the intracellular Ca^2+^ levels, inducing caspase-3 cleavage-mediated apoptosis of cancer cells and suppression of inflammatory responses by inhibiting NF-kB signaling.^99–102^ According to another study, chitooligosaccharides caused inhibitory effects on the proliferation of human rental carcinoma in both *in vitro* and *in vivo* models.^103^

*In vitro* results demonstrated that chitooligosaccharides induced G2/M phase arrest and apoptosis in an ROS-dependent fashion and caused activation of the endoplasmic reticulum (ER) stress signaling pathway. *In vivo* results were consistent with the *in vitro* data, because it was established that chitooligosaccharides repressed tumor growth and ROS accumulation and induced apoptosis mainly via ROS-dependent ER stress pathways.^103^ It was observed that intracellular Ca^2+^ can directly initiate mitochondrial membrane permeabilization, through calcineurin-dependent dephosphorylation of the proapoptotic proteins,^104^ but the coordinated and complex interaction of both Ca^2+^ and ROS appeared to be necessary for the opening of the mitochondrial permeability transition pore and apoptotic (and necrotic) cell death activation.^105^

Although many studies have examined the redox control of Ca^2+^ homeostasis, relatively few studies have investigated this connection specifically as it pertains to carcinogenesis or metastatic progression. In this regard, it is necessary to conduct a deeper research application of natural compounds with redox-modulation activity in cancer therapy as chitosan and its derivatives and to investigate their impact on the intracellular Ca^2+^ homeostasis, ROS generation, and the activation of the human defense system.

## Conclusions and Perspectives

In the present mini-review, the possible redox-regulation potential of chitosan and its derivatives is presented. The considered *in vitro* studies on normal cell lines demonstrated the nontoxic effect of chitosan and its derivatives, due to their action as ROS-scavenging agents and the observed increased levels of intracellular antioxidant enzymes. On the other hand, when applied on cancer cells, the biopolymers have exhibited an anticancer effect, which could be due to the different redox metabolism of the cancer cells as compared with normal ones. The review results displayed increased ROS generation, decreased level of intracellular antioxidant enzymes, depolarization of mitochondrial membrane, caspase activation, and activation of signal transduction pathways for induction of apoptosis. In this study, another aspect of chitosan anticancer activity, due to its immunostimulatory activity, closely related to changed calcium homeostasis in cancer cells, was proposed. In this regard, it is necessary to conduct deeper research for application of natural compounds with redox-modulation activity in cancer therapy, and to investigate their impact on the intracellular Ca^2+^ homeostasis, ROS generation, and the activation of the human defense system.

## Abbreviations Used

AMPK: AMP-activated protein kinase
ER: endoplasmic reticulum
GR: glutathione reductase
iNOS: inducible NO synthase
NADPH: nicotinamide adenine dinucleotide phosphate
ROS: reactive oxygen species
SOD: superoxide dismutase
